# Anakinra for systemic onset juvenile idiopathic arthritis: experience as a second versus first line treatment

**DOI:** 10.1186/1546-0096-9-S1-P190

**Published:** 2011-09-14

**Authors:** S Ricart, J Calzada, V Torrente, R Bou, J Anton

**Affiliations:** 1 Pediatric Rheumatology Unit, Department of Pediatrics. Hospital Sant Joan de Déu, Barcelona, Spain

## Background

Based on the current knowledge of the Systemic onset Juvenile Idiopathic Arthritis (SoJIA) physiopathology, Anakinra is an emergent drug in its treatment. Initially we used it as a second-line treatment, however according with some reports of its efficacy in the induction of remission with an earlier use, we have started to use as a first-line therapy.

## Aim

To describe the response to anakinra in our patients with SoJIA, and to compare the differences in the treatment with anakinra as a first or second-line drug.

## Methods

Retrospective review of the patients treated with Anakinra in our pediatric rheumatology service since 2005. Clinical and analytical data were registered at the beginning of the symptoms, at diagnose and at follow-up (on days 1-3, 7-14, 30 and 3-6 months after starting on Anakinra). SPSS Statistics 19.0 software was used for statistical analysis.

## Results

Among 23 patients treated with Anakinra, in 3 (13%) patients it has been used as a first-line drug without corticosteroids (group A), in 5 (22%) as a first-line with corticosteroids (group B) and in 15 (65%) patients Anakinra was used as a second-line treatment when others DMARDs failed to control the disease (group C). There were no statistical differences in sex, age of diagnose of SoJIA and in the initial dose of Anakinra (median of 2.5 mg/kg/day) between groups. The median time between the initial symptoms of SoJIA and the start of Anakinra was 21 days (A), 43 days (B) and 175 days (C) (p 0.025). At 1-3 days, 7-14 days, 30 days and 3-6 months after starting Anakinra a statistical significant reduction of the values of CRP, ESR, ferritin and white blood cells was observed in all the patients, without statistical differences between groups. The analytical parameter which experienced a faster reduction was CRP with a median of reduction at 1-3 days of 78.92% (A), 75.95% (B) and 83.93% (C). Adverse effects were registered in 5 of 23 patients (21.7%): 2 in group A, 1 in group B and 2 in C. They consist in 3 hypersensitivity reactions (2 in group A, and 1 in group C), elevation of transaminases not explained by other cause in one patient of group B (with normalization after stopping Anakinra), and death due to sepsis in a patient of group C (patient with a Kabuki syndrome and a severe pulmonary affection, alveolar proteinosis). In the group treated exclusively with Anakinra, the treatment was stopped after 110 days in one patient for clinical and analytical remission; in the other two, the initial response was clinically and analytically satisfactory, but Anakinra had to be stopped due to hypersensitivity reactions.

## Conclusions

Based on our data Anakinra could be an effective first-line treatment in SOJIA, alone or in association with steroids, reaching a fast clinical and analytical improvement as well as in the classical second-line use. The predominance of adverse effects in the group treated only with Anakinra should be more investigated due to our small number of patients in this group.

